# MicroRNA-200 and microRNA-30 family as prognostic molecular signatures in ovarian cancer: A meta-analysis

**DOI:** 10.1097/MD.0000000000011505

**Published:** 2018-08-10

**Authors:** Min Shi, Yulan Mu, Hui Zhang, Ming Liu, Jipeng Wan, Xiaoyan Qin, Changzhong Li

**Affiliations:** Department of Obstetrics and Gynecology, Shandong Provincial Hospital Affiliated to Shandong University, Jinan, Shandong, People's Republic of China.

**Keywords:** biomarker, meta-analysis, microRNA, ovarian cancer, prognosis

## Abstract

**Background:**

MicroRNAs (miRs) play a vital role in the occurrence, development, and progression of human cancers, but its role in the prognosis of ovarian cancer is unclear.

**Methods:**

We performed a meta-analysis by searching *PubMed*, *Embase*, and *Web of Science* databases for eligible studies. The pooled hazard ratios (HRs) and 95% confidence intervals (CIs) were used to explore the association between miRs expression and overall survival (OS) and progression-free survival (PFS) on ovarian cancer patients. We also used Kaplan–Meier to analyze the relationship between miRs and OS in OncoLnc dataset.

**Results:**

A total of 15 records were included into the meta-analysis. The expression level of miR-200 family showed significant association with OS (HR = 0.78, 95% CI: 0.64–0.94) and insignificant association with PFS (HR = 0.72, 95% CI: 0.50–1.03). Subgroup analysis revealed that an increased expression level of miR-200c was associated with better OS (HR = 0.59, 95% CI: 0.45–0.74). An increased expression level of miR-200a, miR-200c, and miR-141 was associated with better PFS (miR-200a, HR = 0.59, 95% CI: 0.42–0.75; miR-200c, HR = 0.50, 95% CI: 0.14–0.87, miR-141, HR = 0.38, 95% CI: 0.12–0.63). Similarly, higher expression of miR-30 family was associated with elevated OS/PFS for ovarian cancer (OS, HR = 0.43, 95% CI: 0.13–0.74; PFS, HR = 0.76, 95% CI: 0.64–0.87). The OncoLnc dataset presented that elevated expression level of miR-30d-5p was associated with better OS (n = 470, *P* = .0197).

**Conclusion:**

The meta-analysis reveals that miR-200 family and miR-30 family could be promising prognostic biomarkers of ovarian cancer.

## Abstract 2

Supplemental Digital Content is available in the text

## 1 Introduction

Ovarian cancer represents one of the most common gynecological neoplasms in the world, with an estimated 238,700 new cases and 151,900 deaths in 2012.^[1]^ Although there are many new advances in the understanding of the molecular pathogenesis of ovarian cancer, there is still a lack of effective treatment. Therefore, there is an urgent need to offer more reliable prognostic biomarkers for effectively evaluating the outcomes of this disease and improving the treatment effect. Although a number of prognostic biomarkers have been exploited for ovarian cancer, yet reliably prognostic factors are still relative scanty.^[2]^ As miRs are frequently reported dysregulated in some cancer and often had important roles in the carcinogenesis of various cancers, they seem to be novel and attractive potential indicators for cancer. Some studies have demonstrated that dysregulated expression of miRs can be utilized as a prognostic marker to examine the disease outcome during the treatment of diseases.^[3–7]^ Therefore, effective prognostic markers for ovarian cancer are urgently needed.

MiRs (19–25 nucleotides) are a kind of short noncoding RNAs, which can regulate posttranscriptional expression of target genes.^[8]^ MiR-200, a family of tumor suppressor miRs, consists of miR-141, miR-200a, miR-200b, miR-200c, and miR-429. Study showed that the loss of expression of the miR-200 family members may play an important role in the repression of E-cadherin by zinc finger E-box-binding homeobox 1 (ZEB1) and ZEB2 during epithelial-to-mesenchymal transition (EMT), thereby enhancing migration and invasion during cancer progression.^[9]^ In addition, some studies suggested that the miR-200 family might be serving as a prognostic marker for the treatment outcome in ovarian cancer.^[3,10,11]^ Similarly, the miR-30 family is evolutionarily conserved and consists of 5 members, miR-30a, miR-30b, miR-30c, miR-30d, and miR-30e.^[12]^ Some data had showed that the miR-30 family might be serving as a prognostic marker for the treatment outcome in ovarian cancer.^[13,14]^ However, their conclusions remain controversial. Therefore, we conducted a meta-analysis of studies that have identified a relationship between miR-200 and miR-30 family expression and survival in ovarian cancer.

In our present study, we performed a meta-analysis to provide a better understanding between the expression of miR-200 and miR-30 family and the prognosis in patients with ovarian cancer.

## 2 Materials and methods

### 2.1 Literature search strategy

A computerized literature was performed on *Pubmed*, *Embase*, and *Web of Science* databases for relevant studies that assessed the association between miRs and prognosis in ovarian cancer. All published articles were searched using the following keywords: (microRNA OR miRNA OR miR) AND (ovarian cancer) AND (prognosis OR prognostic OR survival OR outcome OR mortality). The searches were limited to articles published in English. Two investigators (MS and YLM) inspected the titles and abstracts of citations to identify relevant publications and obtained the full texts carefully. We also manually screened the reference lists of retrieved articles in order to identify other relevant studies.

### 2.2 Inclusion and exclusion criteria

Articles were considered eligible if they met all of the following initial inclusion criteria: focused on patients undergoing treatment for ovarian cancer; measured the expression of miRs in blood/tumor samples; had clearly defined the cutoff values; had clearly described detection methods for miRs; analyzed the correlation between OS/PFS and miRs expression; clearly described the follow-up time; clearly described the sample size; clearly described the study population. Articles were excluded following exclusion criteria: were conference records; had sample size <30 cases; calculated HRs based on the combination of multiple miRs; could not be calculated the HRs and 95% CI; or the survival data originated from TCGA dataset. Thereafter, articles that fulfilled all selection criteria were processed for data extraction. Two individual investigators (MS and YLM) independently assessed the eligibility of the retrieved articles. Disagreements were resolved by consensus and consultation with a third researcher (CZL).

### 2.3 Quality assessment

The quality of studies were assessed according to the following checklist based on the proposal by preferred reporting items for systematic reviews and meta-analyses (PRISMA)^[15]^ and reporting recommendations for tumor marker prognostic studies (REMARK):^[16]^ had clearly described study population; had clearly described outcome assessment by representing it in OS or PFS; had clearly defined the measurement methods of miRs (quantitative real-time polymerase chain reaction [qRT-PCR] or in situ hybridization [ISH], etc.); had clear definition of cutoff values; measured the miRs expression level in blood/tumor samples; the follow-up time >60 months; and the sample size >30.

### 2.4 Data extraction

Data were extracted independently by 2 investigators (MS and YLM) who used a standard predefined sheet. The following data were extracted: title; the name of first author; publication year; study design; type of miRs; study population; number of participants; sample types; the measurement methods of miRs expression; cutoff values; follow-up time; HRs together with their 95% CIs and *P* values. If the HRs (95% CIs) and *P* values were not available in the original article, the data were calculated using the Kaplan–Meier curves and the methods illustrated by Parmar et al^[17]^ and Tierney et al.^[18]^ An observed HR >1 and *P* value ≤.05 indicated a worse outcome for the group with elevated miR expression. Conversely, an observed HR<1 and *P* value ≤.05 indicated a worse outcome for the group with decreased miR expression.^[19]^*P* value >.05 indicated no significance.

### 2.5 The validation for the results of meta-analysis

We used Kaplan–Meier and log-rank methods to analyze the relationship between miR-200 family and miR-30 family and OS in OncoLnc dataset (http://www.oncolnc.org/).

### 2.6 Statistical analysis

OS was defined as the time interval between the date of primary surgery and the data of death from any cause.^[20]^ PFS was defined as the time interval between the start of the treatment and the first sign of appearance of relapse or disease progression.^[3]^ Meta-analysis was carried out using the Stata 12.0 software (StatCorp, College Station, TX). A test of heterogeneity was conducted using Higgins *I*^2^ statistic and Cochran *Q* test. *P* value <.05 for *Q* test and *I*^2^ value >50% indicated heterogeneity among studies.^[21]^ The random effect model was applied if the heterogeneity was observed, whereas the fixed-effect model was applied in the absence of between-study heterogeneity. Sensitivity analysis was used by excluding one study by turns and examining the influence of each single study on the combined of HRs. The publication bias was assessed by funnel plots and Egger bias indicator test.

### 2.7 Ethics statement

The Institutional Review Board of Shandong University (Jinan, China) approved this study.

## 3 Results

### 3.1 Selection of studies

**Figure 1:**
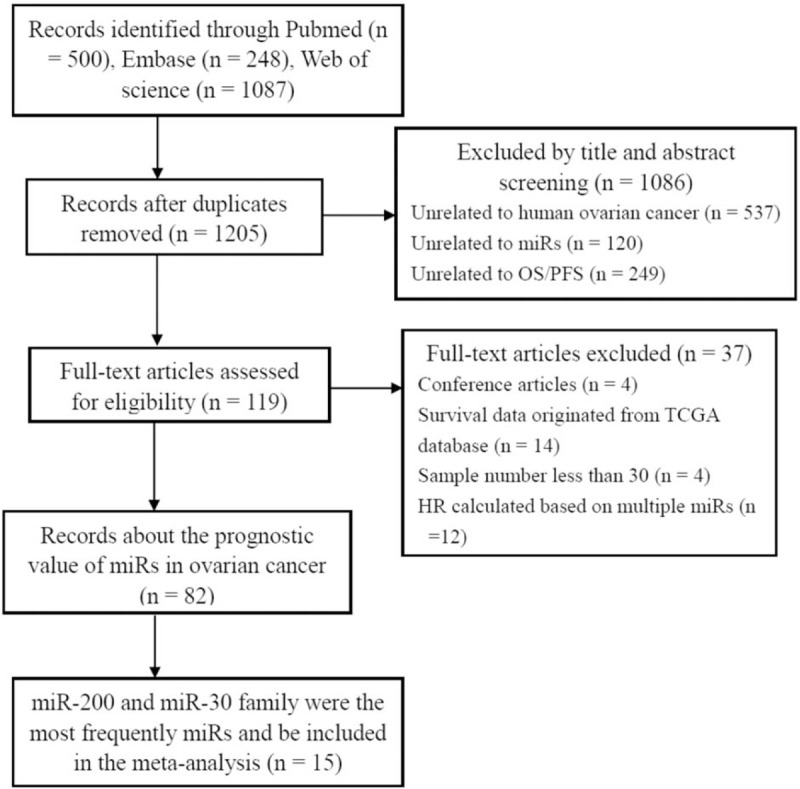
Flow diagram of the study selection procedure. OS = overall survival. PFS = progression-free survival. TCGA = The Cancer Genome Atlas.

A total of 1205 abstracts were found through literature search in *PubMed*, *Embase*, and *Web of Science* databases (Fig. 1). After excluding overlapping abstracts and irrelevant studies, 119 studies were identified as eligible for full-text review. When we checked the full-text, we found that miR-200 family, including miR-200a,^[10,20,22–24]^ miR-200b,^[3,10,22,25–27]^ miR-200c,^[3,10,22,25,27–30]^ miR-141,^[10,22,28,30]^ and miR-429,^[3,10,22]^ were the most frequently miRs with prognostic values in ovarian cancer patients. We also found that miR-30 family, including miR-30a,^[3,13,14,31]^ miR-30b,^[22,24]^ miR-30c,^[24]^ miR-30d,^[3,22,24,32]^ and miR-30e,^[13,14]^ ranked second. So, the association between miR-200 and miR-30 family and the prognosis were included in this meta-analysis.

### 3.2 Characteristics of the included studies

All of the included studies were published recently (2009–2016). Those 15 studies included 1417 patients with ovarian cancer, and sample size ranged 30 from 179 patients. MiRs expression was mainly detected in tissue samples; only 3 studies detected in blood. Table 1 shows the main characteristics of studies included into the meta-analysis. Table 2 shows the related data from the included studies. Studies reported a median of 16 (range: 15–19) items of 20 from the REMARK reporting guidelines (Supplementary Table 1).

**Table 1:**
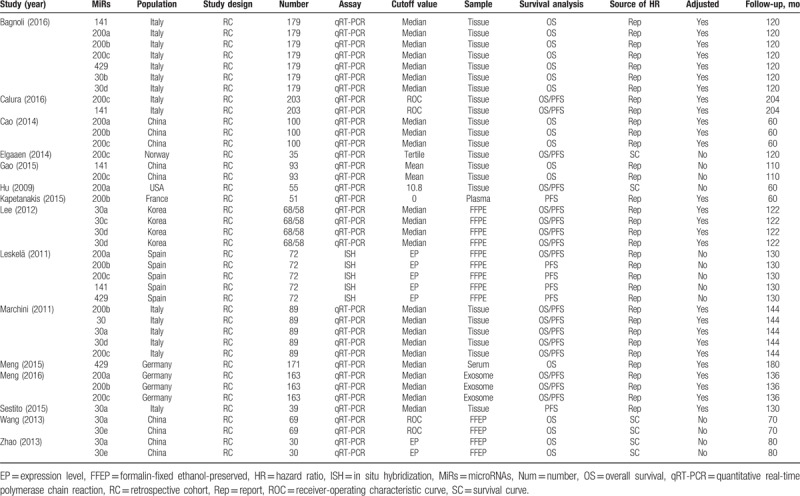
Main characteristics of the eligible studies.

**Table 2:**
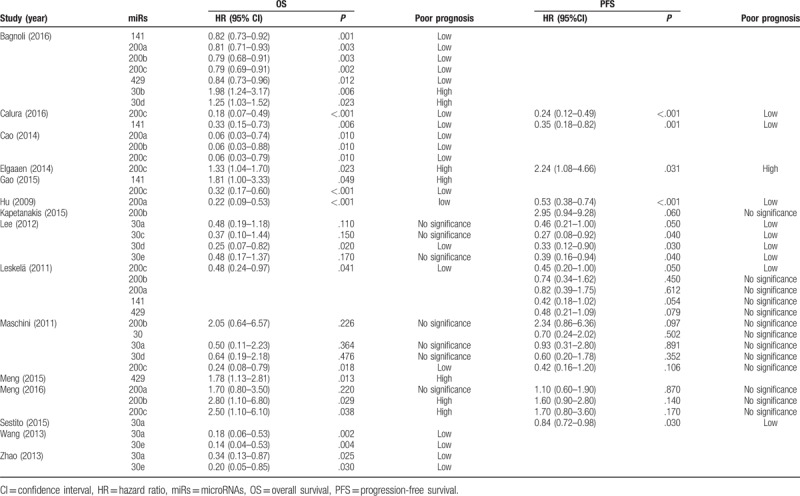
Descriptive characteristics and related data from included studies.

### 3.3 Meta-analysis for miR-200 family

**Figure 2:**
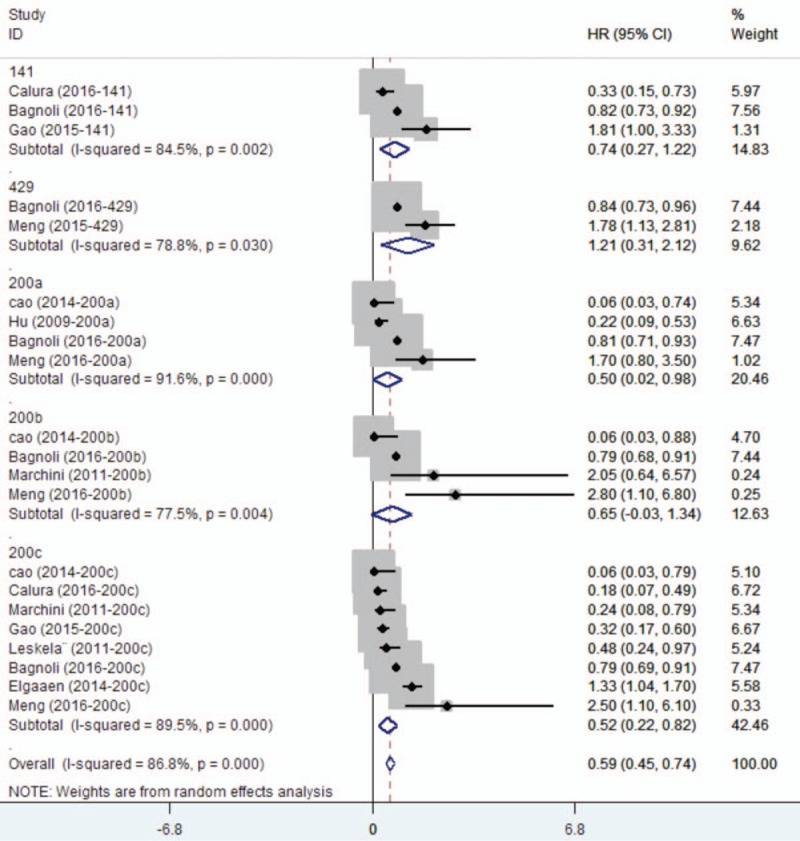
Forest plots of subgroup analysis regarding specific miR-200 family member expression and OS. OS = overall survival.

Eleven studies assessed the association between miR-200 family and survival outcome in ovarian cancer. For miR-200 family, significant interstudy heterogeneity was found (*P* < .05, *I*^2^ = 83.4%), and so we applied the random-effects model (HR = 0.78, 95% CI: 0.64–0.94) (Supplementary Figure 1). Sensitivity analysis by omitting one study by turns showed there was no obvious influence of individual study on the pooled HRs (Supplementary Figure 2). Stratified analysis by miR-200 family member types revealed that elevated expression level of miR-200c was subsequently significantly associated with better OS (HR = 0.59, 95% CI: 0.45–0.74) (Fig. 2). But there was no significant association with respect to miR-200a, miR-200b, miR-141, or miR-429. The Egger test showed no significant publication bias in these studies for miR-200a (*P* = .174), for miR-200b (*P* = .497), for miR-200c (*P* = .220), for miR-141 (*P* = .602), and the funnel plot was showed in Supplementary Figure 3A, 3B, 3C, 3D. Only 2 studies showed the association between miR-429 and ovarian cancer, and the publication bias was not evaluated.

When considering the sample types, the elevated expression of miR-200c was subsequently significantly associated with better OS in tissue (HR = 0.44, 95% CI: 0.27–0.73) (Supplementary Figure 4A). Although the upregulation of miR-200 family indicated a poorer OS in blood (HR = 1.97, 95% CI: 1.42–2.73) (Supplementary Figure 4B), subgroup analysis by study kinds revealed that there was no significantly association between the expression level of miR-200 family and OS (Supplementary Figure 5A and 5B). When we divided miR-200 family into 2 clusters based on chromosomal location, Chr1 (miR-200a, miR-200b, and miR-429) and Chr12 (miR-141 and miR-200c), we found that heterogeneity still exists (Supplementary Figure 5C).

**Figure 3:**
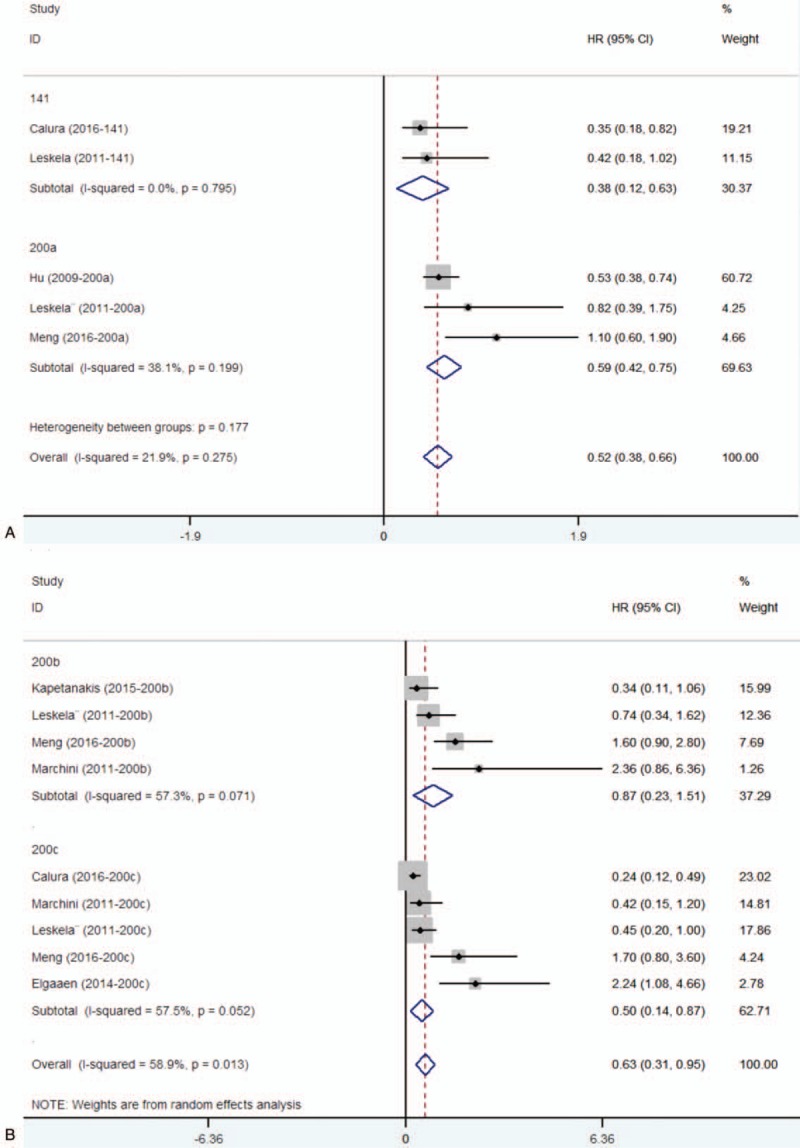
Forest plots of subgroup analysis regarding specific miR-200 family member expression and PFS. (A) miR-141, miR-200a, and PFS. (B) miR-200b, miR-200c, and PFS. PFS = progression-free survival.

We also found that elevated expression level of miR-200 family had no significant association with better PFS (HR = 0.70, 95% CI: 0.43–1.15) (Supplementary Figure 6). Stratified analysis by miR-200 family member types revealed that the upregulated expression of miR-200a, miR-200c, and miR-141 was subsequently significantly associated with better PFS (miR-200a, HR = 0.59, 95% CI: 0.42–0.75; miR-200c, HR = 0.50, 95% CI: 0.14–0.87; miR-141, HR = 0.38, 95% CI: 0.12–0.63) (Figs. 3A and B).

### 3.4 Meta-analysis for miR-30 family

**Figure 4:**
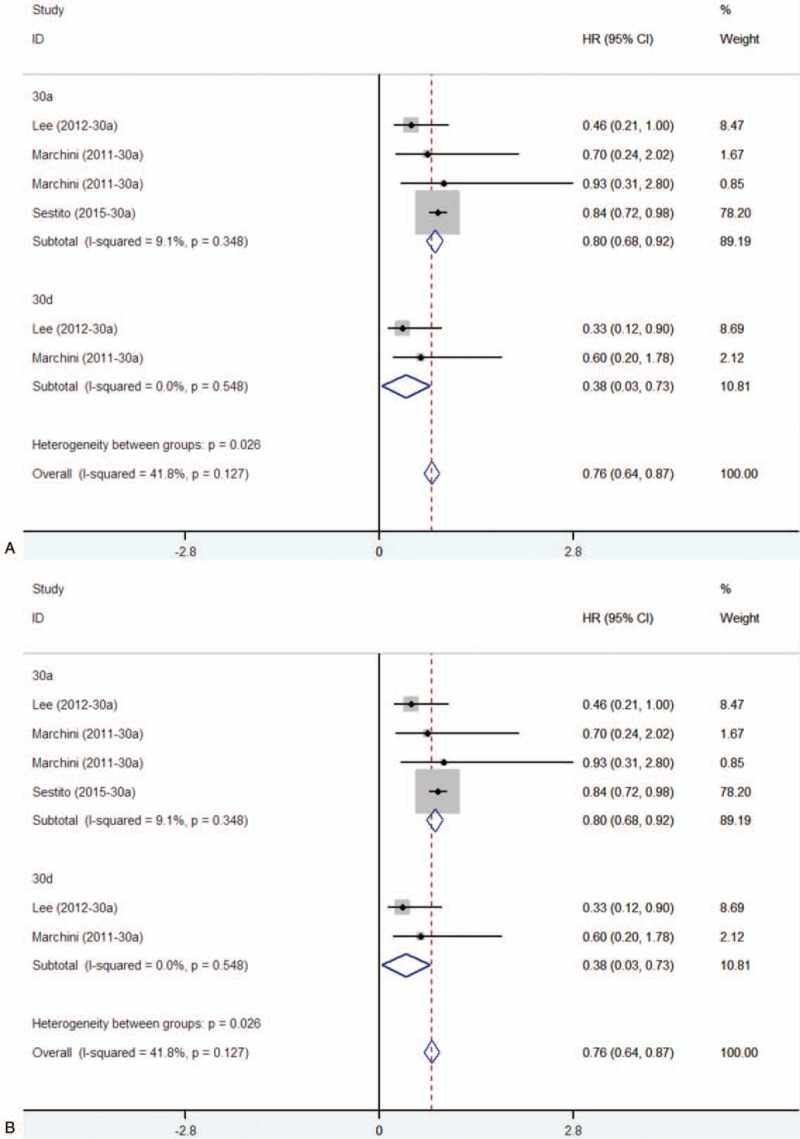
Subgroup analysis regarding specific miR-30 family member expression and OS (A)/PFS (B). OS = overall survival. PFS = progression-free survival.

Six studies had showed that miR-30 family could be a predictor of treatment outcome for ovarian cancer. All of the studies used the tissue as sample. For miR-30 family, significant interstudy heterogeneity was found (*P* < .05, *I*^2^ = 83.7%), and the random-effects model was applied (HR = 0.43, 95% CI: 0.13–0.74) for OS (Fig. 4A). Stratified analysis by miR-30 family member types revealed that elevated the expression levels of miR-30a and miR-30e were subsequently significantly associated with better OS (Fig. 4A). For PFS, no significant interstudy heterogeneity was found (*P* = .127, *I*^2^ = 41.8%), and the fixed-effect model revealed that miR-30 family expression was inversely related with patient's PFS (HR = 0.76, 95% CI: 0.64–0.87) (Fig. 4B). Stratified analysis by miR-30 family member types revealed that elevated the expression level of miR-30a and miR-30d were subsequently significantly associated with better PFS (miR-30a, HR = 0.80, 95% CI: 0.69–0.92; miR-30d, HR = 0.38, 95% CI: 0.03–0.73) (Fig. 4B).

### 3.5 The validation for the results of meta-analysis

**Figure 5:**
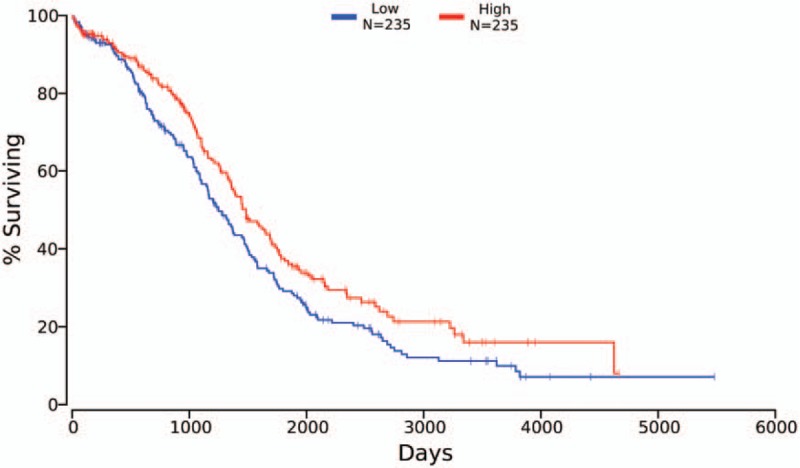
OS curves of 470 ovarian cancer patients for miR-30d-5p low expression (blue line) and high expression (red line). OS = overall survival.

For miR-200 family and miR-30 family, we only found that the elevated expression of miR-30d-5p was associated with better OS (n = 470, *P* = .0197) (Fig. 5). We used median of miR-30d-5p expression as the cutoff value.

## 4 Discussion

It has been shown that miR-200 family were accumulated in ovarian cancer patients. The pooled analysis of studies demonstrated that, for miR-200 family, the improved OS existed only for enhanced expression of miR-200c, which accords with the results by Shi.^[11]^ MiR-200 family members have been reported to regulate EMT by targeting ZEB1 and ZEB2, resulting in dysregulation of the cell–cell adhesion molecule E-cadherin.^[33,34]^ E-cadherin downregulation is clearly important in cancer progression, facilitating cell detachment and metastasis. Furthermore, miR-200 family members target EMT regulators, apparently being important in tumor progression.^[29]^ Studies also found that cells expressing miR-200c played an important role in restoring expression of E-cadherin and altering morphology from mesenchymal to epithelial.^[35]^ Similarly, the pooled analysis of studies demonstrated that, for miR-200 family, the improved PFS existed for elevated expression of miR-200a and miR-141. EPH receptor A2, one of miR-200a targets, promotes tumor growth and predicts poor prognosis for ovarian cancer patients.^[36]^

The pooled analysis of studies demonstrated that higher expression of the miR-30 family significantly improved the OS and PFS in women with ovarian cancer. The subgroup analysis of miR-30 family members revealed that the improved OS existed for enhanced expression of miR-30a and miR-30e. The improved PFS existed for enhanced expression of all miR-30a and miR-30d. Studies showed that activating transcription factor 3 (ATF3), MYC proto-oncogene, and bHLH transcription factor were potential cotargets of miR-30 family, which present as regulators in the different pathways in numerous human cancers.^[14]^ The OncoLnc dataset showed that only elevated expression of miR-30d-5p associated with better OS, which suggesting the potentiality of miR-30d to be used as prognostic biomarker for ovarian cancer. The ten–eleven translocation (TET) family members are new DNA demethylation-related proteins. The study found that TET3 can block transforming growth factor β1 (TGF-β1) by demethylating the miR-30d precursor gene promoter.^[37]^ Studies also showed that miR-30d functioned as a suppressor of ovarian cancer progression by decreasing Snail expression and thus blocking TGF-β1-induced EMT process.^[38]^ The pooled studies can provide a reference for studying the mechanism of ovarian cancer and targeted therapy.

This meta-analysis had several limitations. First, significant heterogeneity existed among the studies. When we analyzed the heterogeneity origin from the study kinds and sample types, the high degree of heterogeneity still exists. Second, the number of studies available was limited. More studies should be conducted to assess these associations in further. Third, circulating markers are more acceptable than tissue markers. More studies may warrant further research to evaluate the prognostic value of miR level in serum.

In conclusion, we found that elevated expression of miR-200 and miR-30 family were indicators of a better treatment outcome in ovarian cancer patients. For further study, investigating the expression of miR-200 and miR-30 family in ovarian cancer may provide a new thinking into cancer prevention and therapeutic strategy.

## Author contributions

**Conceptualization:** Min Shi, Yulan Mu.

**Data curation:** Yulan Mu.

**Formal analysis:** Yulan Mu, Xiaoyan Qin.

**Funding acquisition:** Changzhong Li.

**Investigation:** Yulan Mu, Hui Zhang, Xiaoyan Qin.

**Methodology:** Yulan Mu, Hui Zhang, Xiaoyan Qin.

**Project administration:** Hui Zhang, Ming Liu, Jipeng Wan, Xiaoyan Qin.

**Resources:** Hui Zhang, Ming Liu, Jipeng Wan.

**Software:** Ming Liu, Jipeng Wan.

**Supervision:** Changzhong Li, Min Shi, Ming Liu, Jipeng Wan.

**Validation:** Changzhong Li, Min Shi.

**Visualization:** Min Shi.

**Writing – original draft:** Min Shi.

**Writing – review and editing:** Min Shi.

## Supplementary Material

Supplemental Digital Content
