# Supplementary material for: MicroRNA-200 and microRNA-30 family as prognostic molecular signatures in ovarian cancer: A meta-analysis
Source: Medicine (Baltimore). 2018 Aug 10;97(32):e11505. doi: 10.1097/MD.0000000000011505 (PMC6133642; doi:10.1097/MD.0000000000011505)

Supplementary Table 1. Reporting recommendations for tumour marker prognostic studies (REMARK)

Items	Authors										
	Bagnoli	Calura	Cao	Elgaaen	Gao	Hu	Kapetanakis	Lee	Leskelä	Marchini	Me
**Introduction**											
1.objectives and hypotheses	√		√	√	√	√		√	√	√	√
**Materials and Methods**											
**Patients**											
2.characteristics	√	√	√	√	√	√	√	√	√	√	√
3.treatment methods	√										
**Specimen characteristics**											
4.biological material	√	√	√	√	√	√	√	√	√	√	√
**Assay methods**											
5. method protocol	√		√	√	√		√	√	√	√	√
**Study design**											
6.method of case selection	√	√	√	√	√	√	√	√	√	√	√
7.define clinical and points examined	√	√	√	√	√	√	√	√	√	√	√
8.candidate variable	√										
9.sample size	√	√	√	√	√	√	√	√	√	√	√
**Statistical analysis methods**											
10.statistical methods	√	√	√	√	√	√	√	√	√	√	√
11.cutpoint	√	√	√	√	√	√	√	√	√	√	√
**Results**											
**Data**											
12.number of patients	√	√	√	√	√	√	√	√	√	√	√
13.demographic characteristics	√	√	√	√	√	√	√	√	√	√	√
**Analysis and presentation**											
14. the relation of the marker to standard prognostic variables	√	√	√	√	√	√	√	√	√	√	√
15.univariate analyses	√	√	√	√	√	√	√	√	√	√	√
16.multivariable	√	√	√				√	√		√	√

analyses											
17. effects and confidence intervals	√	√	√		√		√	√	√	√	√
18.further investigations						√					√
**Discussion**											
19.interpret the results	√	√	√	√	√	√	√	√	√	√	√
20.implications for future research	√	√		√	√	√		√	√	√	√

Supplementary Figure 1. Forest plot showing the combined HR from included studies for the association between the expression levels of miR-200 family and OS. OS = overall survival.

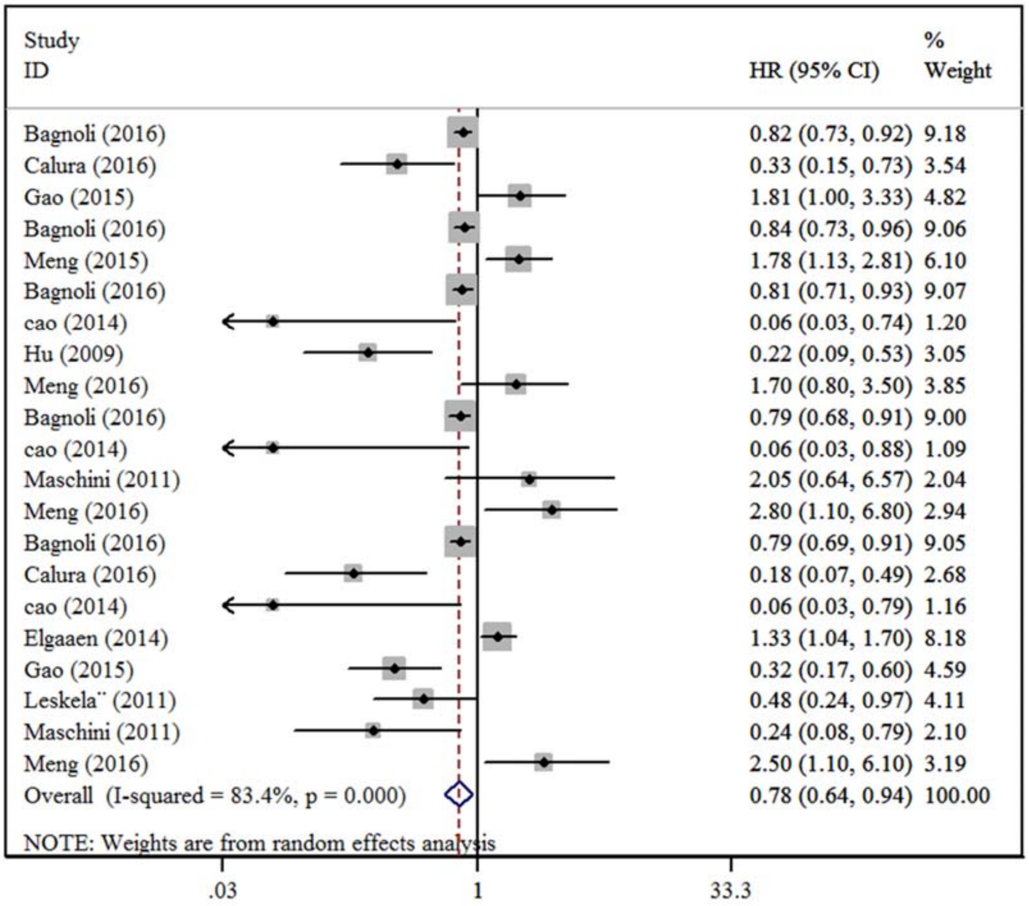

Supplementary Figure 2. Sensitivity analyses of studies regarding miR-200 family expression and OS. OS = overall survival.

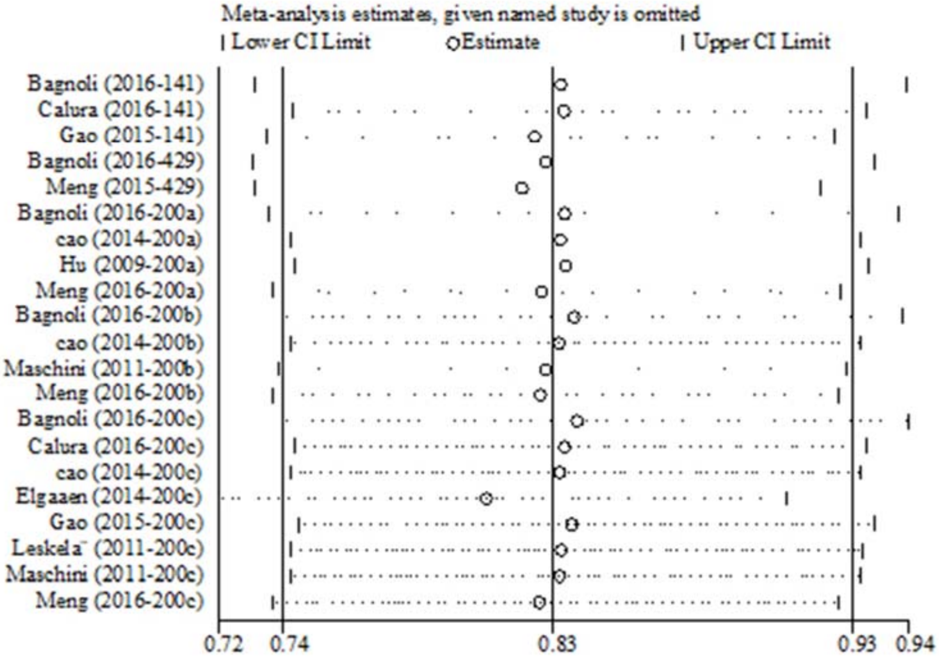

(A)

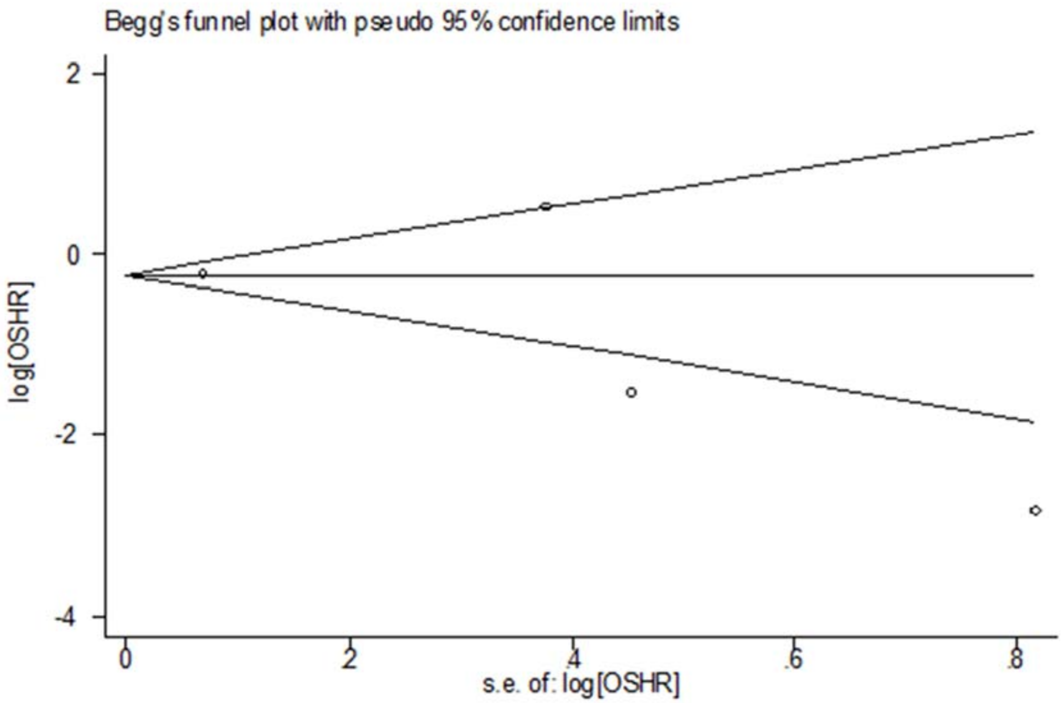

Supplementary Figure 3. Begg's funnel plot of standard error for assessing publication bias. A, miR-200a. B, miR-200b. C, miR-200c. D, miR-141.

(B)

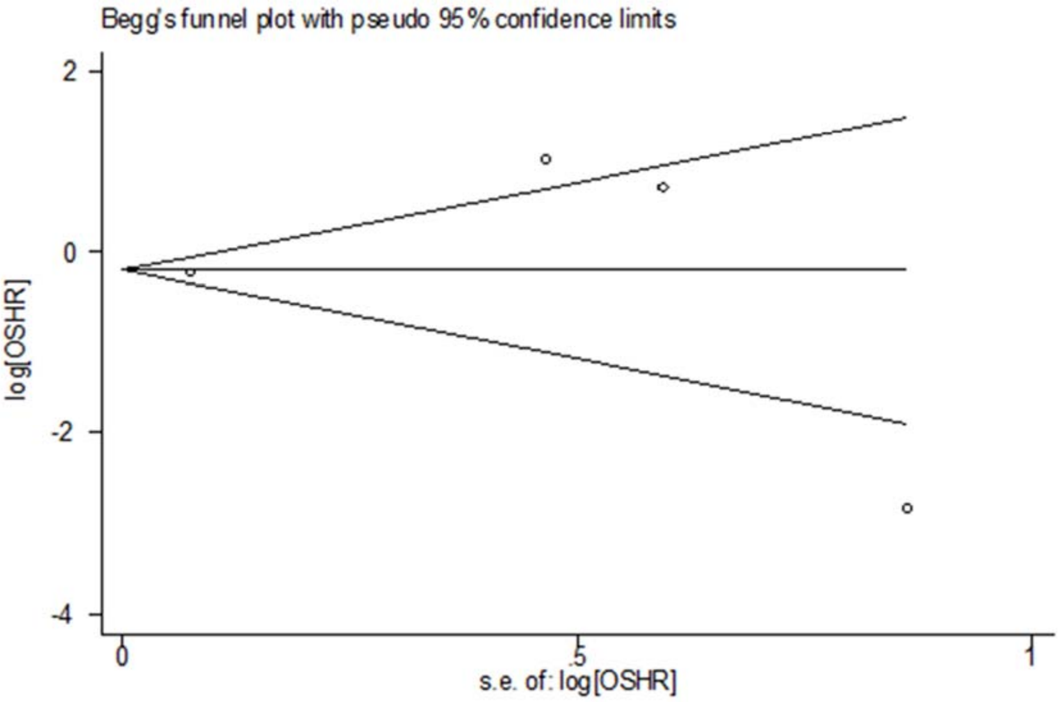
Begg's funnel plot with pseudo 95% confidence limits
2
0
-2
-4
log[OSHR]
0
.5
1
s.e. of: log[OSHR]

(C)

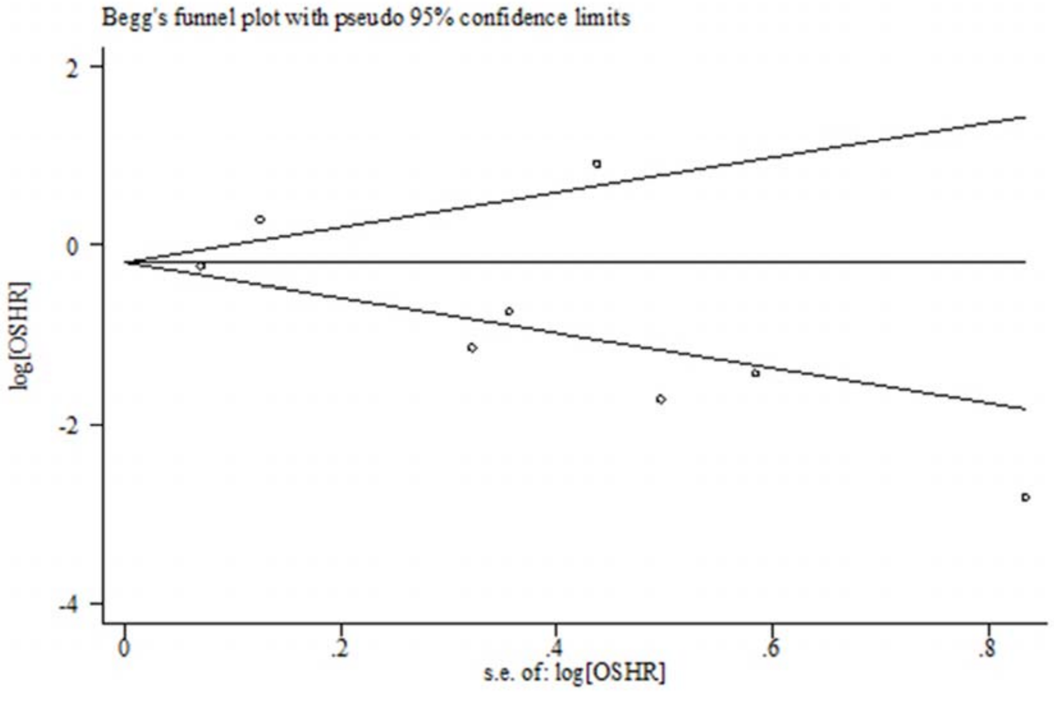
Begg's funnel plot with pseudo 95% confidence limits
2
0
-2
-4
log[OSHR]
0
.2
.4
.6
.8
s.e. of: log[OSHR]

(D)

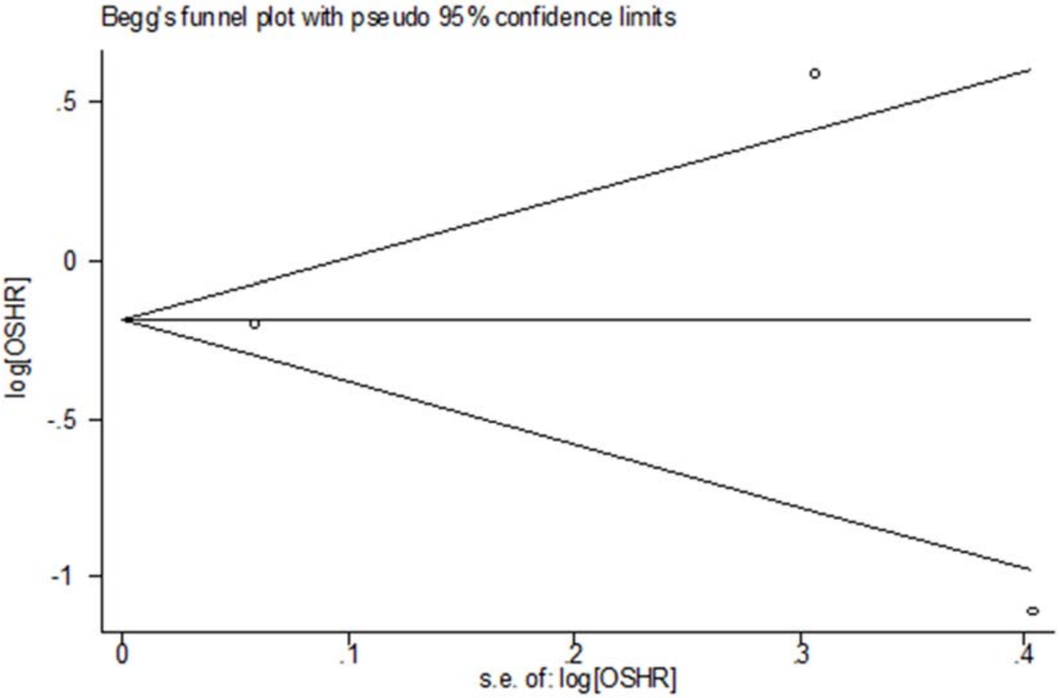

Supplementary Figure 3. Begg's funnel plot of standard error for assessing publication bias. A, miR-200a. B, miR-200b. C, miR-200c. D, miR-141.

Supplementary Figure 4. Forest plots of subgroup analysis by study samples regarding the miR-200 family expression and OS of patients with ovarian cancer. A, the study samples were tissues. B, the study samples were blood.

(A)

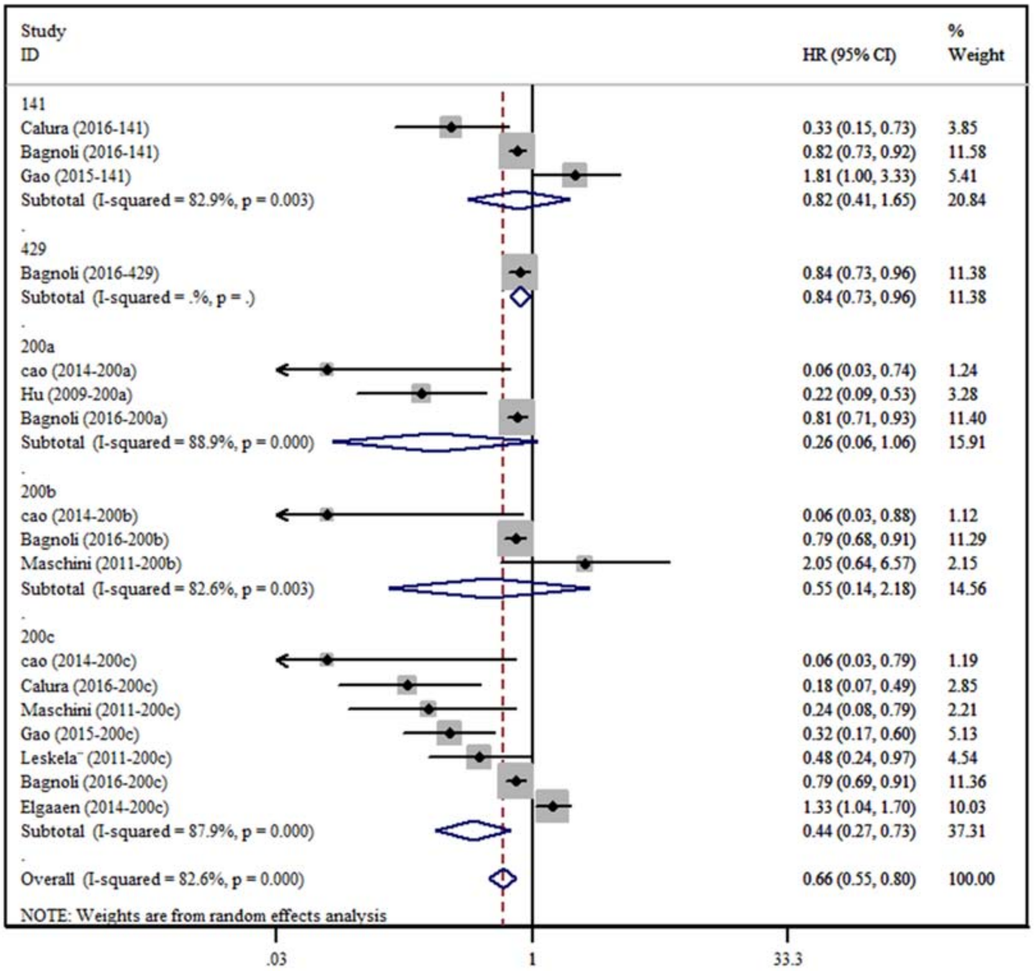

(B)

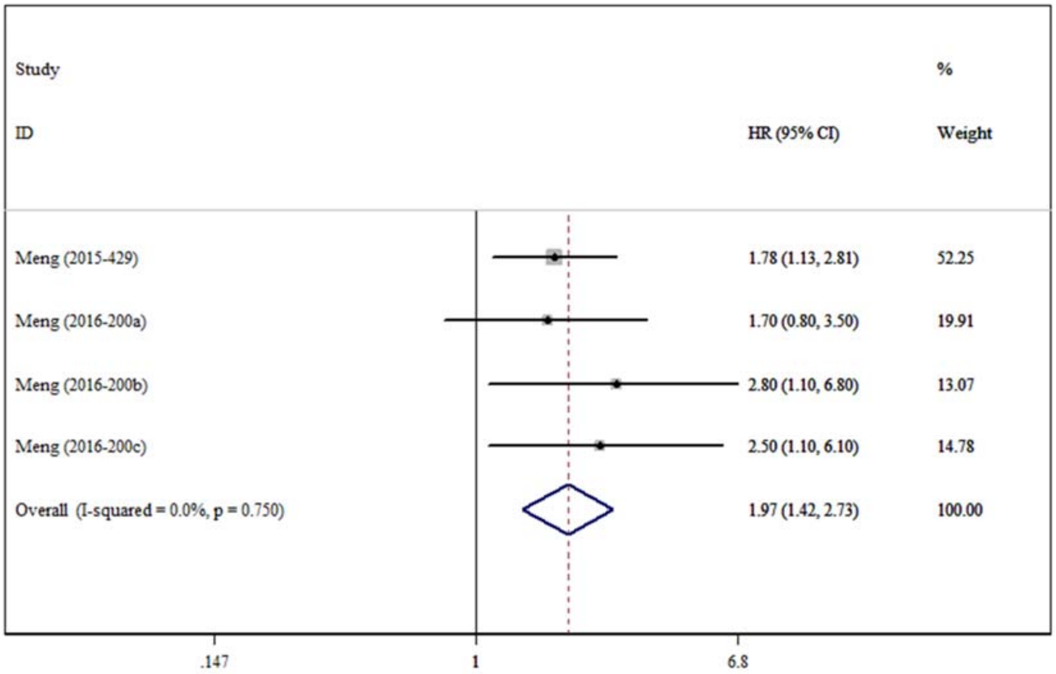

Supplementary Figure 4. Forest plots of subgroup analysis by study samples regarding the miR-200 family expression and OS of patients with ovarian cancer. A, the study samples were tissues. B, the study samples were blood.

Supplementary Figure 5. Forest plots of subgroup analysis by study kinds regarding the miR-200 family expression and OS of patients with ovarian cancer. A, the study kinds were multivariate study. B, the study kinds were univariate study. C, divided miR-200 family into two clusters based on chromosomal location. OS = overall survival.

(A)

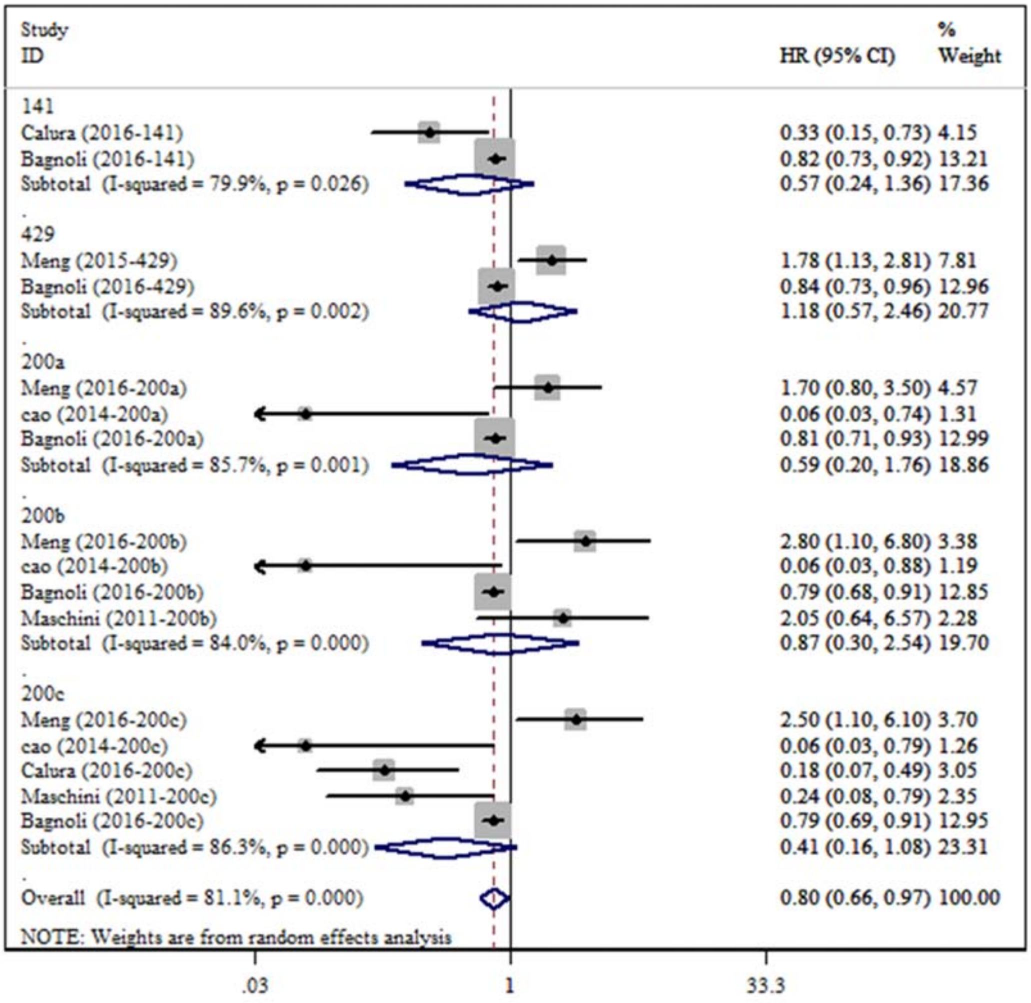

(B)

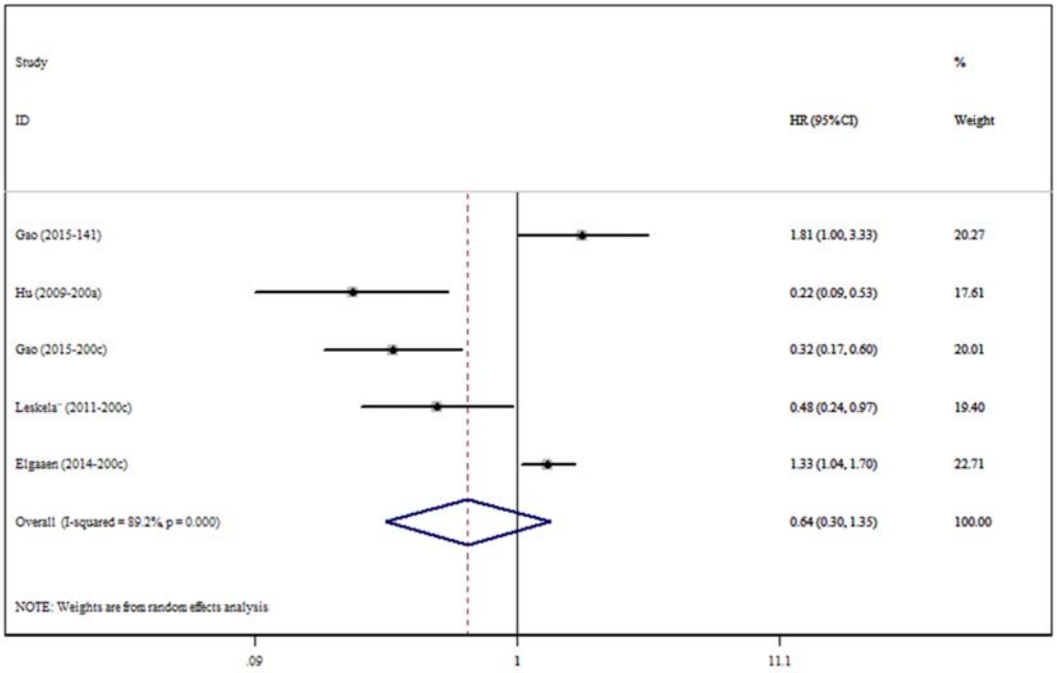

Supplementary Figure 5. Forest plots of subgroup analysis by study kinds regarding the miR-200 family expression and OS of patients with ovarian cancer. A, the study kinds were multivariate study. B, the study kinds were univariate study. C, divided miR-200 family into two clusters based on chromosomal location. OS = overall survival.

(C)

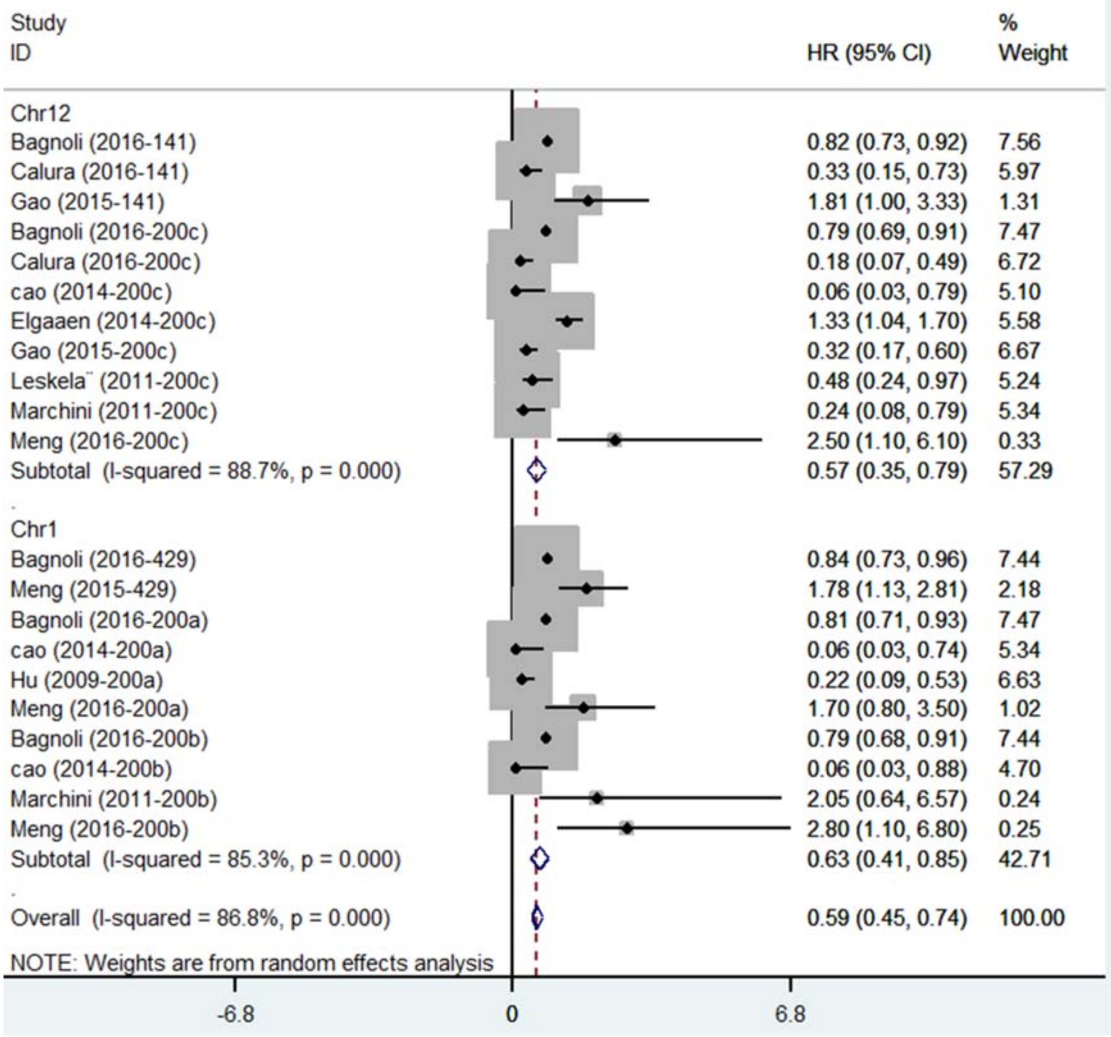
Study
ID
HR (95% CI)
%
Weight
Chr12
Bagnoli (2016-141) 0.82 (0.73, 0.92) 7.56
Calura (2016-141) 0.33 (0.15, 0.73) 5.97
Gao (2015-141) 1.81 (1.00, 3.33) 1.31
Bagnoli (2016-200c) 0.79 (0.69, 0.91) 7.47
Calura (2016-200c) 0.18 (0.07, 0.49) 6.72
cao (2014-200c) 0.06 (0.03, 0.79) 5.10
Elgaaen (2014-200c) 1.33 (1.04, 1.70) 5.58
Gao (2015-200c) 0.32 (0.17, 0.60) 6.67
Leskela¨ (2011-200c) 0.48 (0.24, 0.97) 5.24
Marchini (2011-200c) 0.24 (0.08, 0.79) 5.34
Meng (2016-200c) 2.50 (1.10, 6.10) 0.33
Subtotal (I-squared = 88.7%, p = 0.000) 0.57 (0.35, 0.79) 57.29
Chr1
Bagnoli (2016-429) 0.84 (0.73, 0.96) 7.44
Meng (2015-429) 1.78 (1.13, 2.81) 2.18
Bagnoli (2016-200a) 0.81 (0.71, 0.93) 7.47
cao (2014-200a) 0.06 (0.03, 0.74) 5.34
Hu (2009-200a) 0.22 (0.09, 0.53) 6.63
Meng (2016-200a) 1.70 (0.80, 3.50) 1.02
Bagnoli (2016-200b) 0.79 (0.68, 0.91) 7.44
cao (2014-200b) 0.06 (0.03, 0.88) 4.70
Marchini (2011-200b) 2.05 (0.64, 6.57) 0.24
Meng (2016-200b) 2.80 (1.10, 6.80) 0.25
Subtotal (I-squared = 85.3%, p = 0.000) 0.63 (0.41, 0.85) 42.71
Overall (I-squared = 86.8%, p = 0.000) 0.59 (0.45, 0.74) 100.00
NOTE: Weights are from random effects analysis
-6.8
0
6.8

Supplementary Figure 6. Forest plot showing the combined HR from included studies for the association between the expression levels of miR-200 family and PFS. PFS = progression free survival.

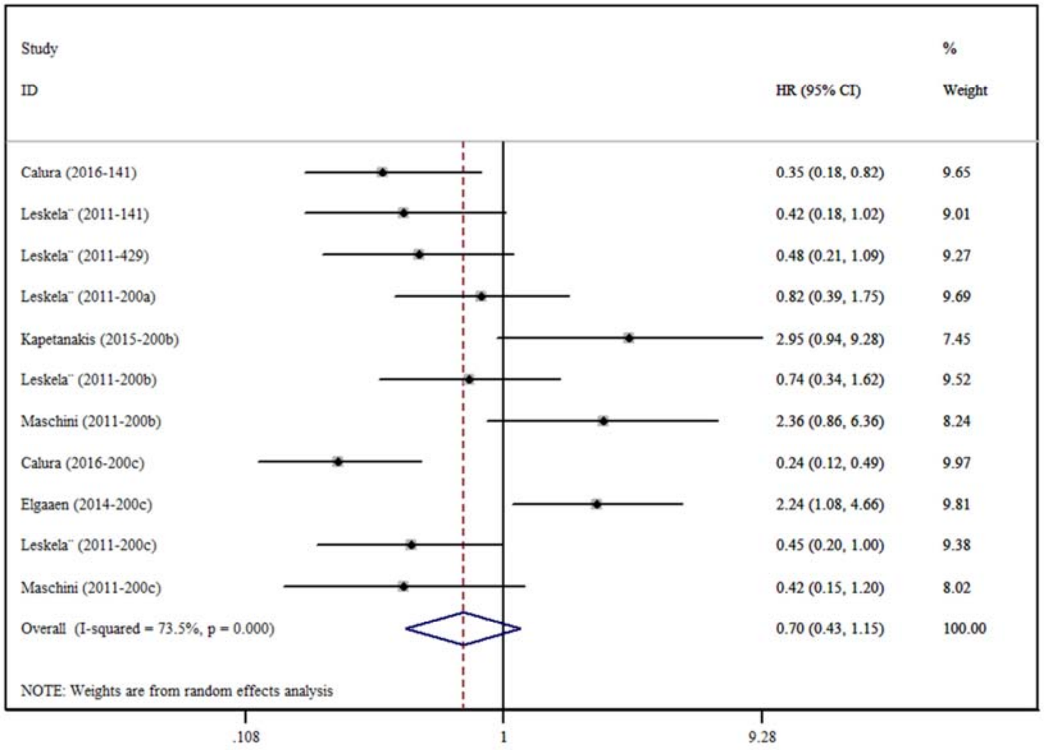

Supplement: Supplemental Digital Content [file medi-97-e11505-s001.pdf]
